# GINS2 affects cell viability, cell apoptosis, and cell cycle progression of pancreatic cancer cells via MAPK/ERK pathway

**DOI:** 10.7150/jca.38386

**Published:** 2020-05-19

**Authors:** Miao Zhang, Saifei He, Xing Ma, Ying Ye, Guoyu Wang, Juhua Zhuang, Yanan Song, Wei Xia

**Affiliations:** 1 Central Laboratory, Seventh People's Hospital of Shanghai University of Traditional Chinese Medicine, Shanghai 200137, China.; 2 Department of Nuclear Medicine, Seventh People's Hospital of Shanghai University of Traditional Chinese Medicine, Shanghai 200137, China.

**Keywords:** Pancreatic cancer, GINS2, MAPK/ERK pathway

## Abstract

**Background and Objective:** GINS complex subunit 2 (GINS2), a member of the GINS complex, is involved in DNA replication. GINS2 is upregulated in a variety of aggressive tumors, such as leukemia, breast cancer, and cervical cancer. However, the role of GINS2 in pancreatic cancer has still remained elusive. In this study, PANC-1 and BxPC-3 cell lines were chosen to perform experiments in vitro. Additionally, the effects of GINS2 interference on the cell viability, cell apoptosis, cell cycle, and tumor growth in nude mice were analyzed.

**Methods:** We utilized pancreatic cancer cell lines that knocked down GINS2 expression using small interference RNA (siRNA) and evaluated GINS2 expression using Western blot analysis. To explore the function of GINS2 in pancreatic cancer cell lines in vitro, MTT assay and flow cytometry were used. Additionally, we investigated the potential mechanism of GINS2 interference by identifying the MAPK/ERK pathway using Western blotting. Finally, PANC-1 cells with GINS2 knockdown were subcutaneously injected into nude mice to evaluate the effects of GINS2 on tumor growth *in vivo*.

**Results:** It was unveiled that GINS2 interference inhibited cell viability, induced cell cycle arrest at G1 phase, and enhanced apoptosis of pancreatic cancer cell lines. Western blot assay indicated that GINS2 interference increased the expression level of Bax, while the expression level of Bcl-2 was remarkably decreased. In addition, the expression levels of CDK4, CDK6, and Cyclin D1 were significantly reduced after treatment with GINS2 siRNA. Furthermore, GINS2 interference drastically attenuated the expression levels of MEK, p-MEK, ERK, and p-ERK, belonging to the MAPK/ERK pathway. The results of an established cancer xenograft model revealed that nude mice transplanted with cells expressing negative control (NC) exhibited larger and heavier tumors, while volume and weight of tumor were remarkably reduced in ones transplanted with cells expressing GINS2 siRNA.

**Conclusions:** GINS2 interference inhibited cell viability, induced cell cycle arrest, and promoted cell apoptosis of pancreatic cancer cell lines via the MAPK/ERK pathway, and our findings may be valuable for treating pancreatic cancer.

## Introduction

Pancreatic cancer is one of the most common causes of cancer-associated mortality worldwide 1,2. The overall 5-year survival rate for pancreatic cancer patients is only 5% 3-5. The major reason for this poor outcome is related to absence of early diagnosis of pancreatic cancer, originating from lack of specific symptoms in the early stages, location of the pancreas deep into the peritoneal cavity, and the early occurrence of metastases 6,7. Thus, understanding the molecular mechanism of oncogenesis is highly essential for clinical management of pancreatic cancer patients.

GINS is one of the core components of the eukaryotic replicative helicase, which is composed of four submits (Sld5, Psf1, Psf2, and Psf3) 8. The GINS complex has been identified to play a pivotal role in the initiation of DNA replication and cell cycle 9. GINS2, also known as Psf2, is a member of the GINS complex, and has been reported to be involved in the tumorigenesis of several types of cancer 10. Previous studies indicated that GINS2 is highly expressed in breast cancer 11, ovarian cancer 12, leukemia 13,14, gliomas 15, and cervical cancer 16. In addition, GINS2 has been shown to promote cell proliferation and desensitize cells to apoptosis. These findings suggest that GINS2 plays a significant role in cancer progression. However, the effects of GINS2 on pancreatic cancer patients have not been reported yet.

The MAPK/ERK pathway (also known as the Ras-Raf-MEK-ERK pathway) is a highly conserved pathway that transfers extracellular signals to cellular proliferation signals 17,18. The MAPK/ERK pathway is an important signal transduction system involved in the regulation of cell proliferation, survival and differentiation 19. However, it has still remained elusive whether GINS2 affects cell viability, cell apoptosis, and cell cycle of pancreatic cancer through the MAPK/ERK pathway.

In the present study, PANC-1 and BxPC-3 cell lines were utilized to perform experiments in vitro. In order to determine the function of GINS2 in human pancreatic cancer, the knockdown of GINS2 was achieved by GINS2 siRNA. Subsequently, the effects of GINS2 interference on the cell viability, cell apoptosis, cell cycle, and tumor growth in nude mice were analyzed. Moreover, bioinformatics analysis was utilized to investigate the association between GINS2 and tumor growth, in order to identify molecular mechanisms on the pathogenesis of pancreatic cancer. Our findings may provide a reliable evidence for the treatment and prognosis of pancreatic cancer patients.

## Materials and methods

### Cell lines and cell culture

Human pancreatic cancer cell lines PANC-1 and BxPC-3 were obtained from the Cell Bank of Type Culture Collection of the Chinese Academy of Sciences (Shanghai, China). PANC-1 and BxPC-3 were cultured in Dulbecco's modified Eagle's medium (DMEM; HyClone Laboratories Inc., Logan, UT, USA) and Roswell Park Memorial Institute (RPMI)-1640 medium (HyClone Laboratories Inc., Logan, UT, USA), respectively, and supplemented with 10% (v/v) fetal bovine serum (FBS; Gibco, New York, NY, USA) and 1% (v/v) penicillin/streptomycin (P/S; Gibco, New York, NY, USA). Cultured cells were maintained at 37 °C with 5% CO2 in a humidified incubator.

### RNA interference

PANC-1 and BxPC-3 were cultured as described. The cells were cultivated in 6-well plates and transfected with GINS2 siRNA or negative control (NC) using Lipofectamine® 2000 Transfection reagent (Invitrogen, Carlsbad, CA, USA) according to the manufacturer's instructions. The GINS2 siRNA sequences were GINS2-1 (5'-GGA UCA UGA ACG AAA GGA ATT-3'), GINS2-2 (5'-GGA CAC UCG UAU AGC CAA ATT -3'), and GINS2-3 (5'-GCU CAA CCA CAU GUA CAA ATT-3'). The NC sequence was 5'-UUC UCC GAA CGU GUC ACG UTT-3'. The above-mentioned siRNA sequences were designed by GenePharma Corporation (Shanghai, China). The effective siRNAs were selected based on the result of Western blotting after 48 h of incubation. After 48 h, the cellular proteins were extracted or certain functional experiments were undertaken (e.g., cell counting kit-8 (CCK-8) test and flow cytometry).

### Western blot analysis

The cultured cells were lysed on ice with radioimmunoprecipitation assay (RIPA) lysis buffer (Beyotime Institute of Biotechnology, Shanghai, China) that vortexed every 15 min for 1 h. The extracts were collected by centrifugation at 12000 rpm for 10 min at 4 °C, followed by transfer of the supernatant to a new tube. The concentrations of proteins were determined using a bicinchoninic acid (BCA) Protein Assay Kit (Beyotime Institute of Biotechnology, Shanghai, China). Proteins (20-50 μg/lane) were loaded on 10% sodium dodecyl sulfate polyacrylamide gel electrophoresis (SDS-PAGE), then transferred onto polyvinylidene difluoride (PVDF) membranes (Bio-Rad Laboratories Inc., Hercules, CA, USA), blocked with 1×PBS and Tween 20 (PBST) containing 5% non-fat milk, and incubated with primary antibodies at 4 °C overnight. The primary antibodies used were anti-human GINS2 rabbit polyclonal antibody, ERK1/2, phospho-ERK1/2, Bax, Bcl-2, CyclinD1, and CDK 4/6 (Cell Signaling Technology, Inc., Danvers, MA, USA). Subsequently, the membranes were incubated with the secondary antibody (Cell Signaling Technology, Inc., Danvers, MA, USA) for 1.5 h at room temperature. Protein bands were visualized on chemical film using enhanced chemiluminescence (ECL) substrates. Glyceraldehyde 3-phosphate dehydrogenase (GAPDH) antibody was served as an internal control. For Western blotting, three independent replicates were performed for each experiment.

### Cell viability assay

Cells were seeded into 96-well plates at a density of 1×10^3^ cells/well. Cell proliferation was assessed using the CCK-8 (Beyotime Institute of Biotechnology, Shanghai, China) assay. After treatment with GINS2 siRNA, cells were grown for 12, 24, 48, and 72 h, and 10 μL CCK-8 was added to each well and incubated for 1 h. Then, the optical density (OD) was measured at wavelength of 450 nm using an enzyme-linked immunosorbent assay (ELISA) plate reader (Promega, Madison, WI, USA). Control cells were treated with cultured media containing 0.15% (v/v) dimethyl sulfoxide (DMSO). At least three independent experiments were carried out.

### Cell apoptosis and cell cycle assays

For cell apoptosis assay, cell pellets were collected after transfection, and stained with the AnnexinV/PI apoptosis kit (Beyotime Institute of Biotechnology, Shanghai, China) according to the manufacturer's instructions. In brief, the cells were trypsinized and re-suspended in 200 µL binding buffer, followed by addition of Annexin V-FITC/PI into the binding buffer; then, the cells were incubated in the dark for 5 min at room temperature. After staining, the cells were analyzed by a flow cytometer (BD Biosciences, San Jose, CA, USA). Data were analyzed by FlowJo V7 software.

For the cell cycle assay, cell pellets were collected and fixed in 70% ethanol at 4℃ overnight after transfection, and treated with 5 ug/ml RNase A at 37 °C for 30 min. After that, they were stained with propidium iodide (PI) solution (BD Biosciences, San Jose, CA, USA) for 30 min at room temperature, and the cells were analyzed by a flow cytometer (BD Biosciences, San Jose, CA, USA). The percentage of cells in G0/G1, S, and G2/M phases was determined by using FlowJo V7 software.

### Microarray and bioinformatics assays

Total RNA was extracted using TRIzol reagent in NC group (n=3) and GINS2 siRNA group (n=3). RNA quality and concentration were determined by an Agilent 2100 Bioanalyzer system (Agilent Technologies, Inc., Santa Clara, CA, USA) and a NanoDrop 2000 spectrophotometer (Thermo Fisher Scientific, Waltham, MA, USA). The qualified samples were analyzed on GeneChip PrimeView Human Gene Expression Array (Thermo Fisher Scientific, Waltham, MA, USA). Differentially expressed genes were selected with a cut-off P-value of less than 0.05 based on statistical analysis and a two-fold change cut-off value. Those differentially expressed genes obtained from the microarray analyses were uploaded onto Ingenuity Pathway Analysis (IPA; Ingenuity Systems, Redwood City, CA, USA), and a core biologic pathway analysis was undertaken to identify molecular pathways.

### *In vivo* xenograft studies in mice

Four-week-old female BALB/c nude mice were obtained from the Laboratory Animal Center of Chinese Academy of Sciences (Shanghai, China) and fed under specific-pathogen-free (SPF) conditions. Mice were randomly divided into two groups, including control (NC) group and GINS2 knock-down (KD) group. Then, PANC-1 cells and KD cells (density, 1 × 10^7^ cells/well) were subcutaneously injected into the right hind limbs, respectively. Tumor growth was monitored by measurement of the length and width weekly, and the tumor volume was calculated using the following equation, (L ×W^2^)/2.

### Statistical analysis

Data were processed using SPSS 16.0 software (IBM, Armonk, NY, USA). Additionally, data were presented as mean ± standard deviation (SD), and analyzed using the Student's t-test or one-way analysis of variance (ANOVA). P<0.05 was considered statistically significant.

## Results

### GINS2-specifc siRNA transfection downregulates GINS2 expression in pancreatic cancer cells

**Figure 1:**
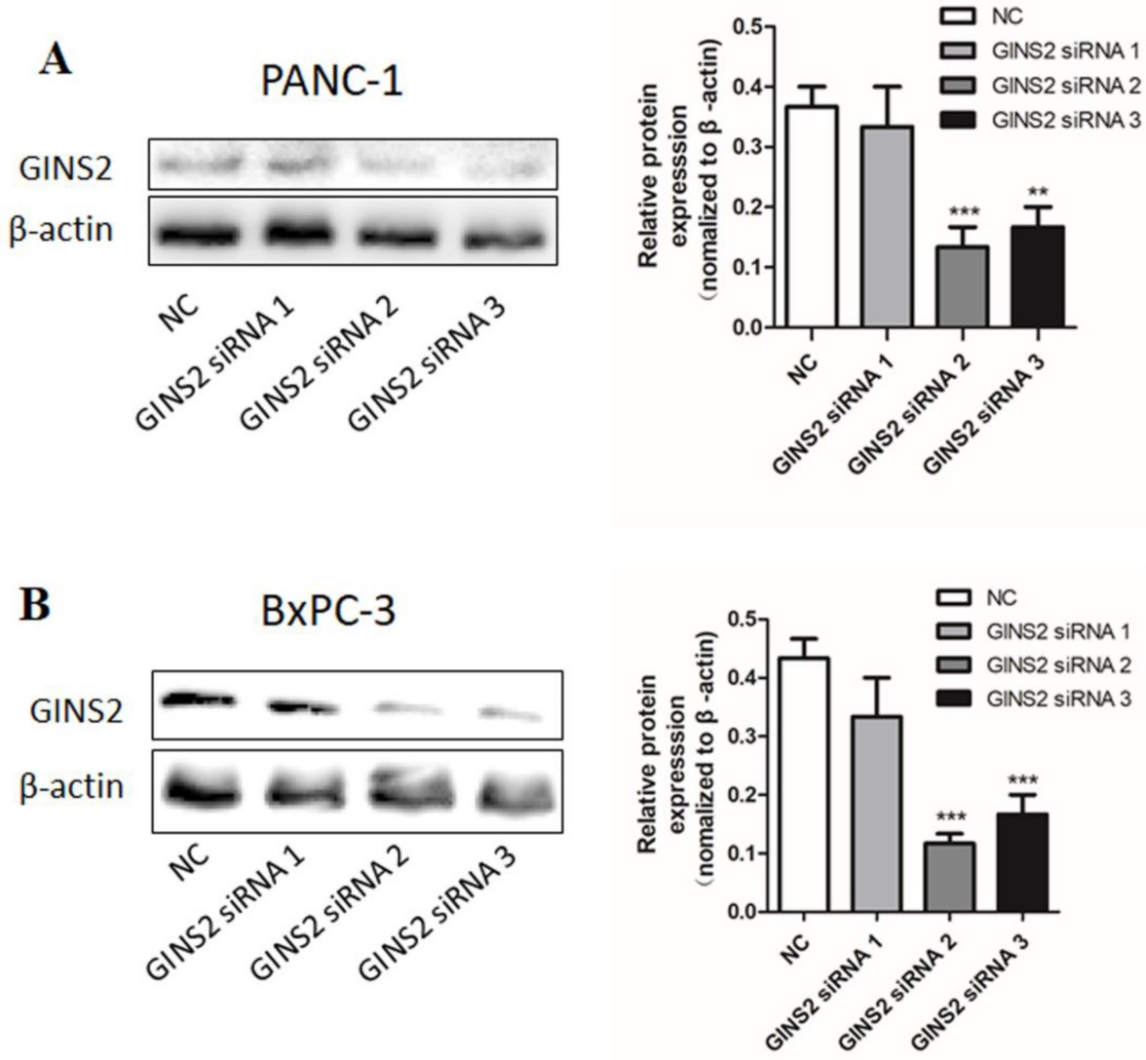
** The expression of GINS2 in PANC-1 and BxPC-3 after transfection of specific GINS2 siRNA.** (**A and B**) Western blot analysis showed the expression levels of GINS2. Error bars represent the standard deviation. siRNA, small interfering RNA; NC, negative control. Values were expressed as mean ± standard deviation (n=3) (* P<0.05, ** P<0.01, ***P<0.001 vs. NC).

For the purpose of studying the role of GINS2 in pancreatic cancer, GINS2 siRNA was prepared and transfected into pancreatic cancer cells. Negative siRNA transfected into pancreatic cancer cells was used as NC, and untransfected pancreatic cancer cells were used as a blank control. The protein expression was analyzed by Western blotting. As shown in Fig. 1, GINS2 expression in pancreatic cancer cells transfected with siRNA was significantly lower compared with NC. These results indicated that GINS2 siRNA was effective in silencing of GINS2 protein expression. Subsequent experiments on the effects of GINS2 knockdown should be carried out using the effective GINS2 siRNA in pancreatic cancer cells.

### GINS2 interference inhibited cell viability in pancreatic cancer cells

**Figure 2:**
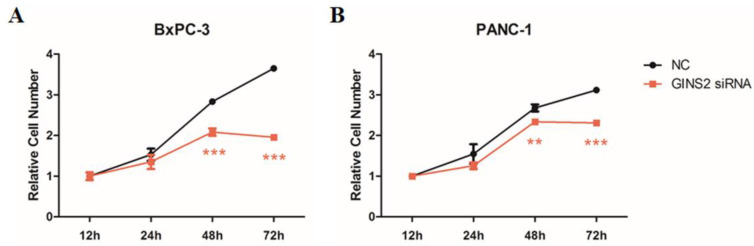
** GINS2 interference inhibited cell viability in pancreatic cancer cells. (A and B)** After transfection of GINS2 siRNA, cell viability was measured by MTT assay in BxPC-3 and PANC-1 cells at 12, 24, 48, and 72 h. The absorbance was measured at OD of 450 nm by using a microplate reader. Data were expressed as mean ± standard deviation (n=3) (* P<0.05, ** P<0.01, ***P<0.001 vs. NC group).

To assess the effects of GINS2 interference on cell viability of pancreatic cancer cells, MTT assay was performed. Results showed that in BxPC-3 cells (Fig. 2A) and PANC-1 cells (Fig. 2B), the absorbance increased from 12 to 72 h in NC group, while that increased from 12 to 48 h and decreased from 48 to 72 h in GINS2 siRNA group. At 48 and 72 h, the number of pancreatic cancer cells in the GINS2 siRNA group was noticeably lower than that in NC group. The above-mentioned findings indicated that GINS2 interference could inhibit cell viability in pancreatic cancer cells.

### GINS2 interference induced cell cycle arrest in pancreatic cancer cells

**Figure 3:**
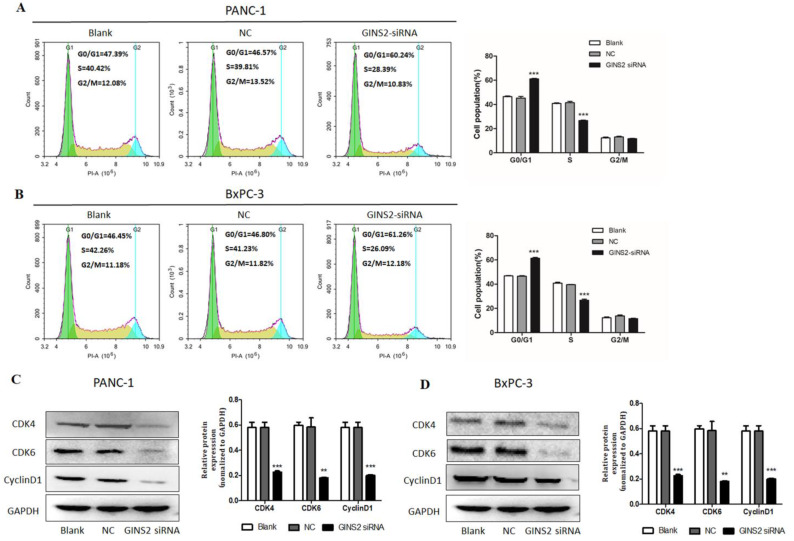
** GINS2 interference induced cell cycle arrest in pancreatic cancer cells. (A and B**). After GINS2 interference, flow cytometry revealed cell cycle condition in PANC-1 and BxPC-3 cells. **(C and D).** Western blot analysis showed the expressions of CDK4, CDK6, and Cyclin D1 with interference of GINS2. Data were presented as mean ± standard deviation (SD) of three independent experiments (* P<0.05, ** P<0.01, ***P<0.001 vs. NC group).

To confirm the role of GINS2 interference in cell cycle, flow cytometry was conducted. It was unveiled that compared with the NC group, GINS2 interference caused a significant increase in the percentage of cells in G0/G1 phase, with a concomitant decrease in the percentage of cells in S phase in both PANC-1 and BxPC-3 cells (Fig. 3A and 3B). Thus, GINS2 interference induced cell cycle arrest in G1 phase. In addition, Western blot analysis was undertaken to assess the protein expressions of CDK4, CDK6, and Cyclin D1. As illustrated in Fig. 3C and 3D, the expressions of CDK4, CDK6, and Cyclin D1 were significantly decreased following the interference of GINS2.

### GINS2 interference induced cell apoptosis in pancreatic cancer cells

**Figure 4:**
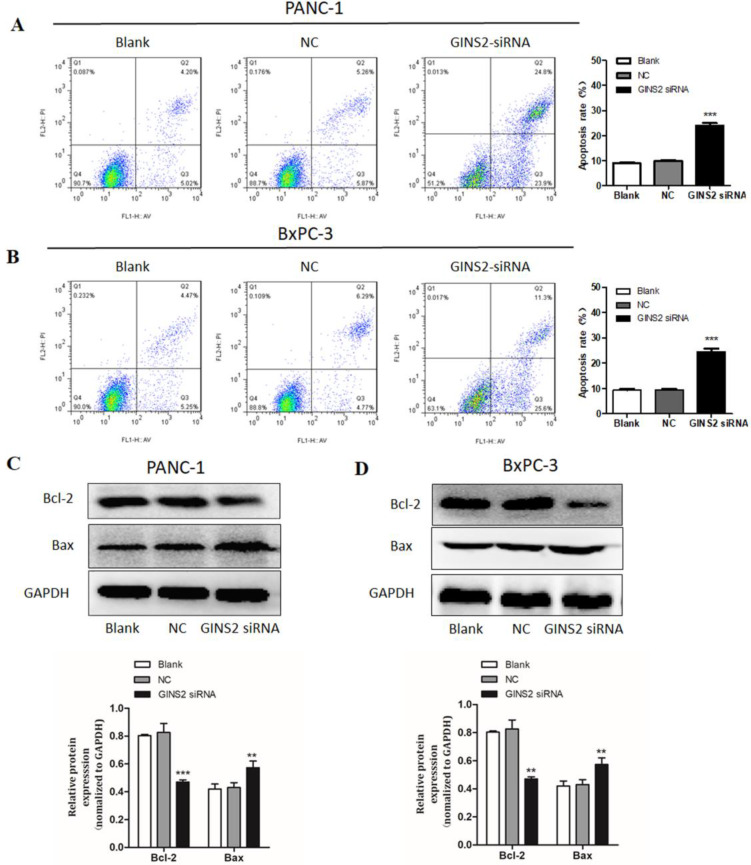
** GINS2 interference induced cell apoptosis in pancreatic cancer cells.** (**A and B**). After treatment with GINS2 siRNA for 48 h, cells were stained with Annexin-V/FITC and PI, and flow cytometry assessed the percentage of apoptotic cells. (**C and D**). Western blot analysis was used to detect the expression levels of Bax and Bcl-2. Data were presented as the mean ± standard deviation (SD) of three independent experiments. FITC, fluorescein isothiocyanate; PI, propidium iodide (* P<0.05, ** P<0.01, ***P<0.001 vs. NC group).

We next evaluated the effects of GINS2 on cell apoptosis in pancreatic cancer cells. Using flow cytometry, the proportion of apoptotic cells was assessed. Our results uncovered that GINS2 interference noticeably promoted the cell apoptosis. As displayed in Fig. 4, the percentage of apoptotic cells was markedly higher in both PANC-1 and BxPC-3 cells compared with NC cells (Fig. 4A and 4B). Furthermore, Western blot analysis disclosed that GINS2 interference elevated the expression level of Bax in both PANC-1 and BxPC-3 cell lines. In contrast, the expression level of Bcl-2 was significantly decreased (Fig. 4C and 4D).

### GINS2 interference influenced the MAPK/ERK pathway

**Figure 5:**
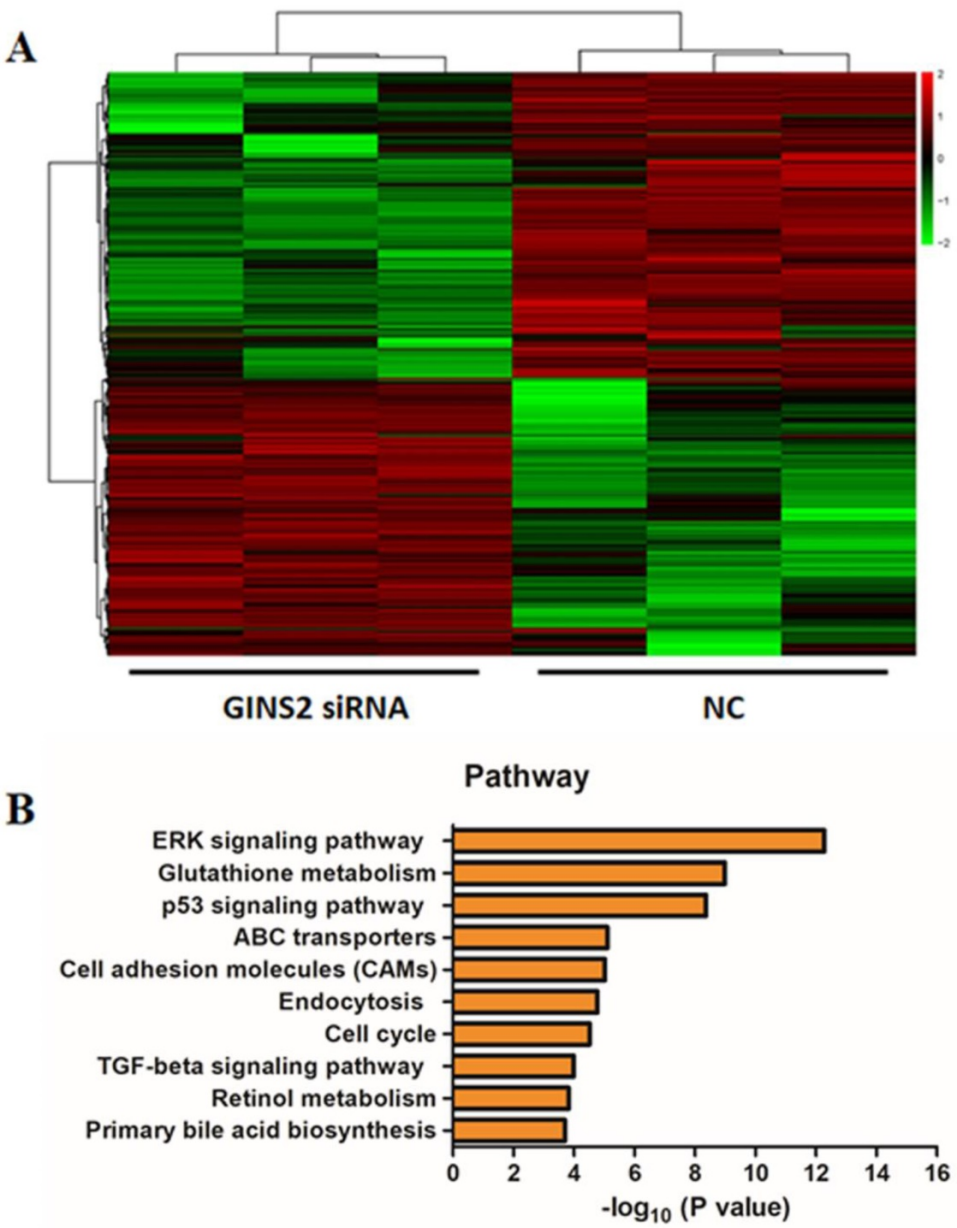
** GINS2 interference influenced the MAPK/ERK pathway. (A)** The heatmap of differentially expressed genes for NC group and GINS2 siRNA group. Red represents up-regulated genes, and green represents down-regulated genes. Upper tree structure is listed according to the sample characteristics, and left tree structure is listed according to the gene characteristics. There is a higher similarity between the adjacent samples or genes.** (B)** The signaling pathway analysis between NC group and GINS2 siRNA group.

In order to confirm the mechanisms of GINS2 interference inhibiting cell proliferation and promoting apoptosis in pancreatic cancer cells, we detected the gene expression profiling of NC group and GINS2 siRNA group in PANC-1 cells. Compared with NC group, a great number of genes were up-regulated or down-regulated. As depicted in Fig. 5A, red represents up-regulated genes, and green shows down-regulated genes. Subsequently, the significantly altered signaling pathways were identified by IPA, and it was revealed that the value of -log10 (P value) for MAPK/ERK pathway was maximum (Fig. 5B). These outcomes indicated that GINS2 may influence the function of pancreatic cancer cells by regulating MAPK/ERK pathway.

**Figure 6:**
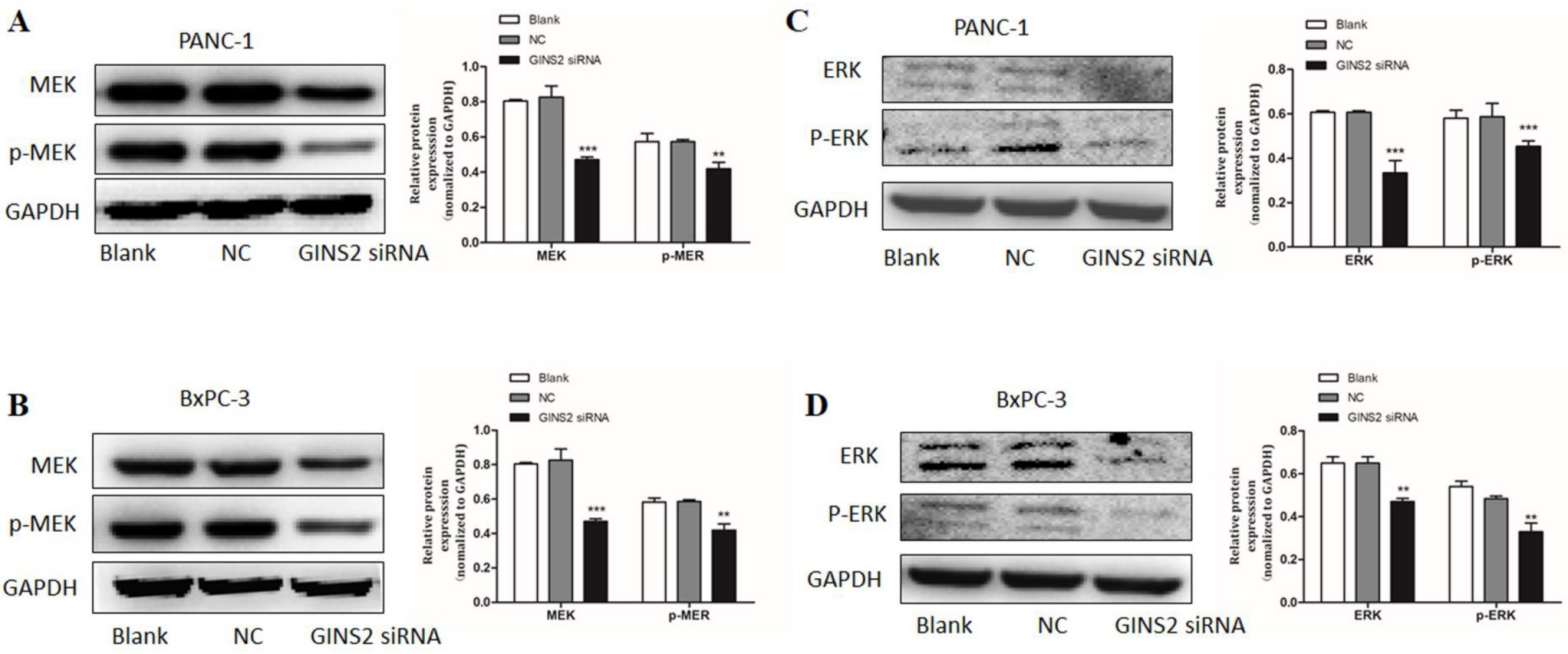
** GINS2 interference affected the biological behaviors of pancreatic cancer cells via the MAPK/ERK pathway. (A-D).** Western blot analysis uncovered that GINS2 interference reduced the expression levels of ERK and p-ERK, MEK and p-MEK in PANC-1 and BxPC-3 cell lines. Data were expressed as the mean ± standard deviation (SD) of three independent experiments (* P<0.05, ** P<0.01, ***P<0.001 vs. NC group).

Furthermore, the expressions of ERK1/2, p-ERK1/2, MEK, and p-MEK were detected by Western blotting. Following the interference of GINS2 by siRNA, the expression levels of ERK1/2 and p-ERK1/2 were decreased compared with the NC in both PANC-1 and BxPC-3 cell lines (Fig. 6C and 6D). Moreover, the expression levels of MEK and p-MEK were remarkably decreased (Fig. 6A and 6B). Therefore, the GINS2 interference suppressed the expression level of ERK, inhibited the phosphorylation of ERK, and altered downstream protein levels, including apoptosis-related proteins and cell cycle-related proteins. Altogether, our data indicated that GINS2 affects the cell proliferation, apoptosis and cell cycle in pancreatic cancer cells via MAPK/ERK pathway.

### GINS2 interference suppressed tumor growth *in vivo*

**Figure 7:**
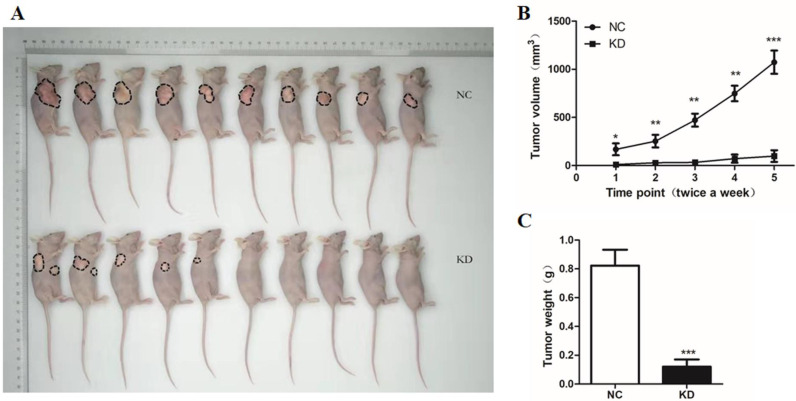
** GINS2 interference suppressed tumor growth *in vivo*. (A)** Subcutaneous injection of NC and siGINS2 PANC-1 cells to detect the effects of GINS2 knockdown. **(B)** Tumor growth curve of cancer xenograft model in nude mice. After 11 weeks, tumor volume was calculated using the formula v=0.5*ab^2^ (a and b represent the longer and shorter diameters of the tumor, respectively) at 3-day and 4-day intervals for two weeks.** (C)** Statistics for the tumor weight. Data were expressed as mean ± standard deviation (SD) (n=10 for each group) (* P<0.05, ** P<0.01, ***P<0.001 vs. NC group).

To further explore the effects of GINS2 knockdown, we established cancer xenograft model in nude mouse by subcutaneous injection of NC and siGINS2 PANC-1 cells, respectively. The results revealed tumor growth for 11 weeks. After 11 weeks, tumor volume was measured at 3-day and 4-day intervals for two weeks. After 13 weeks, mice were sacrificed. We found that the nude mice transplanted with cells expressing NC exhibited larger and heavier tumors, while volume and weight of tumor were remarkably reduced in cells expressing siGINS2 (Figure 7A-C). The above-mentioned findings demonstrated that GINS2 interference could dramatically inhibit tumor growth.

## Discussion

Pancreatic cancer is known as a malignant tumor with high mortality rate even in presence of improved treatment methods 6. Cell apoptosis and cell cycle of tumor are processes of dynamic changes, which are closely associated with dynamic circulation of cytoskeleton 20. Thus, exploring the mechanisms of cell apoptosis and cell cycle in pancreatic cancer is highly urgent for the treatment of pancreatic cancer patients.

DNA replication is an essential event for all living organisms, and thus the basic mechanism is conserved from bacteria to eukaryotes. Genomic DNA replication must be executed accurately and only once during the S-phase of the cell cycle. GINS2, as an important subunit of GINS complexes, mediates the initiation of DNA replication in eukaryotic cells 21. Previous studies reported that high GINS2 expression is associated with several types of malignant cancer, and is involved in the regulation of cell apoptosis, cell cycle, and signaling pathways 8. Therefore, GINS2 was considered as a potential prognostic marker and therapeutic target for these tumors. However, to the best of our knowledge, the association between GINS2 and the progression of pancreatic cancer has still remained obscure.

Apoptosis is a process of programmed cell death, and the underlying mechanism is mainly controlled by physiological or pathologic factors 22,23. To date, numerous scholars were concentrated on molecular mechanism of cell apoptosis, and apoptosis-related proteins were identified as well. These included anti-apoptotic proteins, such as Bcl-2, Bcl-x, Bcl-XL, and pro-apoptotic proteins (e.g., Bax, Bak, and Bid). Among these proteins, Bcl-2 and Bax highly influence cell apoptosis 24,25. In the present study, knockdown of GINS2 inhibited cell viability and promoted cell apoptosis in PANC-1 and BxPC-3 cells. Furthermore, we detected the expression levels of apoptosis-related proteins, and the results revealed that GINS2 interference markedly decreased the expression level of Bcl-2, while increased the expression level of Bax. Our data indicated that GINS2 interference inhibited cell viability and promoted cell apoptosis by increasing the expression level of Bax and decreasing the expression level of Bcl-2 in PANC-1 and BxPC-3 cells.

Since GINS2 mediates the initiation of DNA replication in eukaryotic cells, which is essential for S-phase progression and cell division, GINS2 is more likely to participate in the regulation of cell cycle progression 26. In addition, previous studies indicated that Cyclin D1 is a key protein in cell cycle regulation 27-29. As requisite functional partner kinases of Cyclin D1, suppression of Cyclin-dependent kinases 4 and 6 (CDK4/CDK6) activity successfully blocks Cyclin D1-mediated cell cycle progression, making these protein kinases attractive therapeutic targets 30,31. In the current study, flow cytometry uncovered that the proportion of cells in G0/G1 phase was significantly increased following GINS2 interference. Additionally, GINS2 inhibition resulted in decreased expression levels of CDK4, CDK6, and Cyclin D1, leading to cell cycle arrest in G1 phase.

MAPK cascade is a critical pathway for tumor cell proliferation, differentiation, apoptosis, and resistance to drug therapy. It is currently known to comprise four sub-pathways: ERK, JNK, BMK, and and p38. The ERK pathway is an important signal transduction system for the regulation of cell proliferation, survival and differentiation 32-34. Activation of ERK pathway can promote cell proliferation, differentiation, and cellular survival 35,36. To further confirm our hypothesis, we performed microarray, bioinformatics, and Western blotting assays to indicate whether GINS2 affects the ERK pathway to alter the biological behaviors of pancreatic cancer cell lines. Our findings unveiled that the ERK signaling pathway was significantly altered after GINS2 inference. Additionally, the expression levels of ERK, p-ERK, MEK, and p-MEK were remarkably reduced. The above-mentioned results demonstrated that Bax, Bcl-2, CDK4/6, and Cyclin D1 are functional proteins of the ERK pathway that can affect cell survival and cell cycle. This was consistent with our results that increased apoptotic rate and cell cycle arrest might be caused by GINS2 interference. Taken together, our outcomes indicated that GINS2 influences the cell viability, cell apoptosis, and cell cycle of pancreatic cancer cell lines via the MAPK/ERK pathway.

## Conclusions

**Figure 8:**
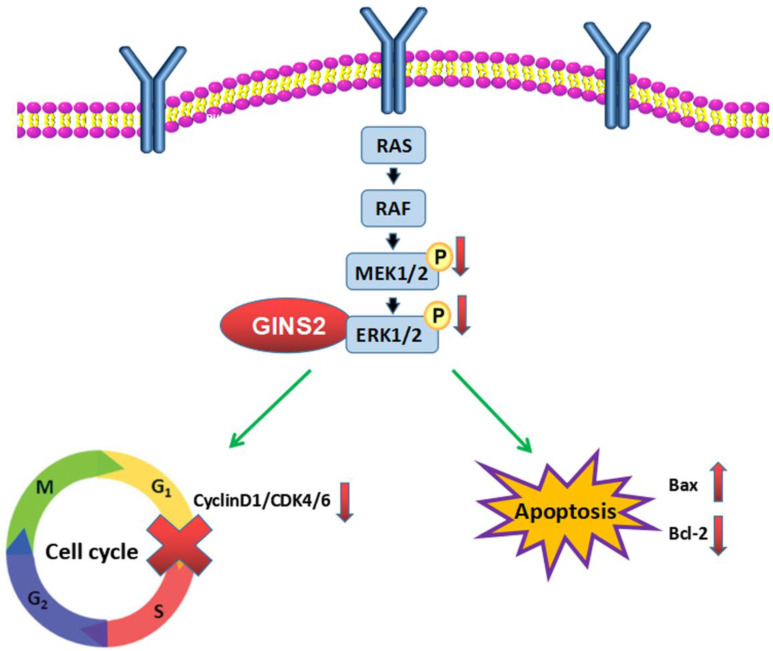
** GINS2 interference induces apoptosis and cell cycle arrest.** GINS2 silencing induces DNA damage and inhibits the activation of MAPK/ERK signaling pathway, then influences cell proliferation, cell apoptosis and cell cycle of pancreatic cancer cell lines.

In summary, we demonstrated that GINS2 interference inhibited the cell viability, promoted cell apoptosis, and induced cell cycle arrest in pancreatic cancer cell lines via the MAPK/ERK pathway (Fig. 8). The mechanism underlying this phenomenon indicated that GINS2 may be highly significant for the treatment of pancreatic cancer.
